# Therapeutic Effect of Large Channel Endoscopic Decompression in Lumbar Spinal Stenosis

**DOI:** 10.3389/fsurg.2021.603589

**Published:** 2021-06-18

**Authors:** Fei-Long Wei, Ming-Rui Du, Tian Li, Kai-Long Zhu, Yi-Li Zhu, Xiao-Dong Yan, Yi-Fang Yuan, Sheng-Da Wu, Bo An, Hao-Ran Gao, Ji-Xian Qian, Cheng-Pei Zhou

**Affiliations:** ^1^Department of Orthopedics, Tangdu Hospital, Fourth Military Medical University, Xi'an, China; ^2^School of Basic Medicine, Fourth Military Medical University, Xi'an, China

**Keywords:** lumbar spinal stenosis, outcomes, safety, large channel, endoscopic decompression

## Abstract

**Background:** Percutaneous endoscopic decompression (PED) is a minimally invasive surgical technique that is now used for not only disc herniation but also lumbar spinal stenosis (LSS). However, few studies have reported endoscopic surgery for LSS. Therefore, we conducted this study to evaluate the outcomes and safety of large channel endoscopic decompression.

**Methods:** Forty-one patients diagnosed with LSS who underwent PED surgery were included in the study. The estimated blood loss, operative time, length of hospital stay, hospital costs, reoperations, complications, visual analogue scale (VAS) score, Oswestry Disability Index (ODI) score, Japanese Orthopaedic Association (JOA) score and SF-36 physical-component summary scores were assessed. Preoperative and postoperative continuous data were compared through paired-samples *t*-tests. The significance level for all analyses was defined as *p* < 0.05.

**Results:** A total of 41 consecutive patients underwent PED, including 21 (51.2%) males and 20 (48.8%) females. The VAS and ODI scores decreased from preoperatively to postoperatively, but the JOA and SF-36 physical component summary scores significantly increased. The VAS (lumbar) score decreased from 5.05 ± 2.33 to 0.45 ± 0.71 (*P* = 0.000); the VAS (leg) score decreased from 5.51 ± 2.82 to 0.53 ± 0.72 (*P* = 0.000); the ODI score decreased from 52.80 ± 20.41 to 4.84 ± 3.98 (*P* = 0.000), and the JOA score increased from 11.73 ± 4.99 to 25.32 ± 2.12 (*P* = 0.000). Only 1 patient experienced an intraoperative complication (2.4%; dural tear), and 1 patient required reoperation (2.4%).

**Conclusions:** Surgical treatment for LSS is to sufficiently decompress and minimize the trauma and complications caused by surgery. This study did not reveal any obvious shortcomings of PED and suggested PED is a safe and effective treatment for LSS.

## Introduction

Degenerative lumbar spinal stenosis (LSS) is characterized by changes in the spinal structure (such as facet joints and ligaments) due to aging, resulting in a reduction in the diameter of the spinal canal (1). LSS is the most common spinal pathology in the elderly population, and the number of patients who need to undergo surgery for the disease has increased (2–4). In the United States, the prevalence of relatively narrow LSS (i.e., 12 mm tube diameter) is as high as 22.5% in the general population, and that of absolute stenosis (i.e., 10 mm tube diameter) is as high as 7.3% (5). These figures increase sharply with age, reaching 47.2 and 19.4%, respectively, among people aged 60 years or older (6). LSS greatly reduces patient quality of life (7).

Minimally invasive surgery techniques are becoming increasingly important in spinal surgery to protect the multifidus muscle, a stabilizer for the spine and locomotor actions (2, 8, 9). At present, endoscopic surgery, a minimally invasive surgery technique, is considered to be an extension of alternative to spinal surgery (10). The surgical indications for endoscopic spine surgery are still increasing due to the release of practical and reliable clinical results (11, 12). Spinal endoscopy is now used to treat not only disc herniation but also LSS (13). Previously, a key obstacle was the need to remove enough bone and the ligamentum flavum under continuous visualization to achieve decompression (14). Advances in technology have made it possible to treat LSS with percutaneous endoscopic decompression (PED) (14, 15).

Although many surgical techniques are available for the treatment of lumbar spinal stenosis, there is little evidence to support this rapidly developing surgical technique, and clinicians often rely on their own opinions and experience (16–18). Few studies have investigated endoscopic surgery for LSS, and their evaluation indicators were relatively simple (19–21). Therefore, we conducted this study to evaluate the efficacy and safety of endoscopic surgery for LSS. In addition, this is the first study to systematically evaluate the application of large channel endoscopy in LSS.

## Methods

### Patient Selection

**Figure 1:**
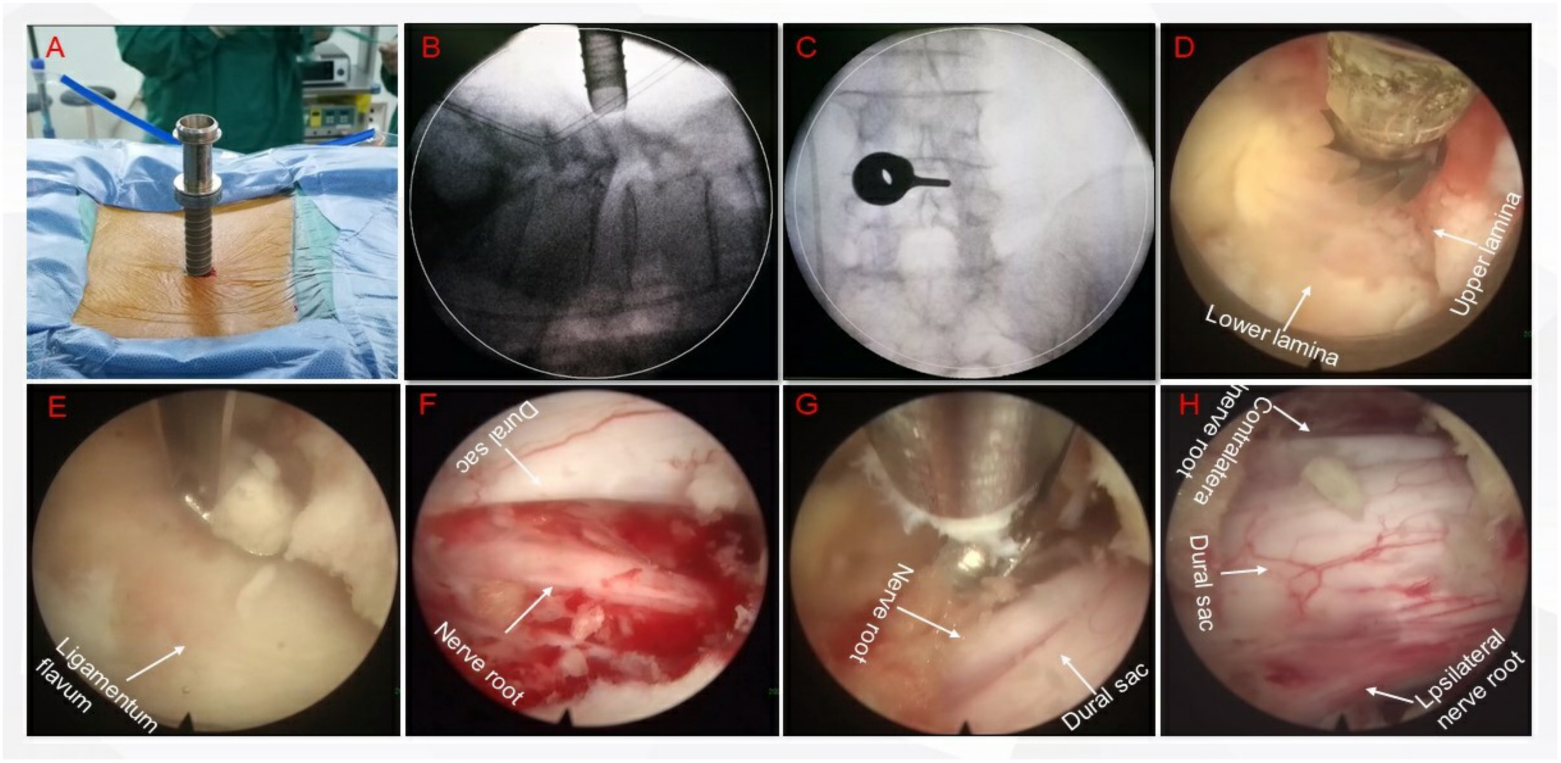
A representative case. **(A)** Place the working channel; **(B)** Lateral X-ray fluoroscopy confirms that the channel is facing the intervertebral space; **(C)** Positive X-ray fluoroscopy confirms that the channel is located inside the facet joint; **(D)** Clean up the soft tissue on the surface of the lamina, confirm the lamina space and upper and lower lamina; E. Remove the lamina and expose the ligamentum flavum; **(F)** Decompression of the nerve root on the same side, ranging from the initial segment of the nerve root, down to the inner edge of the pedicle, explore the Ipsilateral nerve root canal to achieve at least 270° decompression of the nerve root canal; **(G)** Decompression of the contralateral nerve root; **(H)** After the decompression, the dural sac and bilateral nerve roots are visible.

This was a retrospective study. The study protocol was approved by the hospital ethics committee and performed according to the Declaration of Helsinki. Between January 2012 and December 2018, 41 patients diagnosed with LSS who underwent PED were included in the study. The inclusion criteria were as follows: (1) patients with LSS due to neurogenic claudication; (2) Central stenosis or lateral stenosis who need surgery; (3) Low-grade (Meyerding grade 1 or 2) isthmic spondylolisthesis or degenerative spondylolisthesis; (4) patients with imaging findings consistent with the symptoms. The exclusion criteria included trauma, active infection, malignant tumors, spinal deformity, previous lumbar fusion, multi-segment fusion, multi-level, high-grade (Meyerding grade 3 or 4) isthmic spondylolisthesis or degenerative spondylolisthesis; obvious lumbar instability in the surgical segment (the change of cobb angle in hyperextension and flexion is >11° or displacement is >3 mm). A representative case is shown in Figure 1.

### Surgical Procedure

The patients were treated with large-channel endoscopic decompression. The PED operation was performed with bilateral decompression through a unilateral approach. After general anesthesia, each patient was placed in the prone position, and then, the operating table was adjusted to expand the lumbar lamina space. The positioning point was located at the midpoint of the interlaminar space of the facet joint under X-ray. Then, a 15 mm incision was made at the positioning point; the skin and fascia were cut, and they were expanded step by step with a 3rd grade cannula. The depth of the expansion cannula was confirmed under fluoroscopy without breaking the ligament flavum. After the position of the cannula was confirmed to be satisfactory, the working cannula was inserted, and the expansion cannula was removed; the spinal endoscope was connected and inserted. First, the soft tissues on the lamina and ligamentum flavum were cleaned endoscopically. Bony decompression was performed using a high-speed drill under direct endoscopic vision, and then, the ligamentum flavum was removed, completing ipsilateral decompression. Then, the cannula was tilted to remove the contralateral ligamentum flavum and part of the medial bone of the upper articular process to complete contralateral decompression. After the exploration step showed that the extent of decompression was sufficient, the working sleeve was pulled out, and finally, the wound was sutured.

### Outcome Measures

The blood loss, operative time, length of hospital stay, costs, reoperation rate and complications were assessed. We recorded the visual analogue scale (VAS), Oswestry Disability Index (ODI), Japanese Orthopaedic Association (JOA) and SF-36 physical component summary scores of the patients before surgery and at 2 and 3 years after surgery.

### Statistical Analysis

The statistical analyses were performed by SPSS (version 23.0; IBM, Chicago, IL). Preoperative and postoperative continuous data were compared through paired-samples *t*-tests. The significance level for all analyses was defined as *p* < 0.05.

## Results

Forty-one patients were included in this study, including 21 (51.2%) males and 20 (48.8%) females. The mean age was 56.76 ± 13.35 years. The patients had a mean body mass index of 25.34 ± 3.10 kg/m^2^. The most common surgical segment in both groups was L4/5. The mean operative time was 113.41 ± 28.69 min (60–150 min); the volume of intraoperative blood loss was 121.78 ± 82.03 mL (10–300 mL); the length of hospital stay was 10.34 ± 2.84 days (6–23 days); and the total cost was 3.57 ± 0.45 ten thousand yuan (2.89–4.62 ten thousand yuan) (Table 1).

**Table 1:** Clinical characteristics of included patients.

**Variables**	**PED**
	**(*N* = 41)**
Age	56.76 ± 13.35
Gender	
Male	21 (51.2%)
Female	20 (48.8%)
BMI	25.34 ± 3.10
Smoker	
Yes	7 (17.1%)
No	34 (82.9%)
Hypertension	
Yes	5 (12.2%)
No	36 (87.8%)
Diabetes	
Yes	3 (7.3%)
No	38 (92.7%)
Operative segments	
1	37 (90.2%)
2	4 (9.8%)
3	0 (0.0%)
Operative time(min)	113.41 ± 28.69
Blood loss (mL)	121.78 ± 82.03
Hospital stay	10.34 ± 2.84
Cost	35735.68 ± 4493.08

*Plus–minus values are means ±SD*.

**Figure 2:**
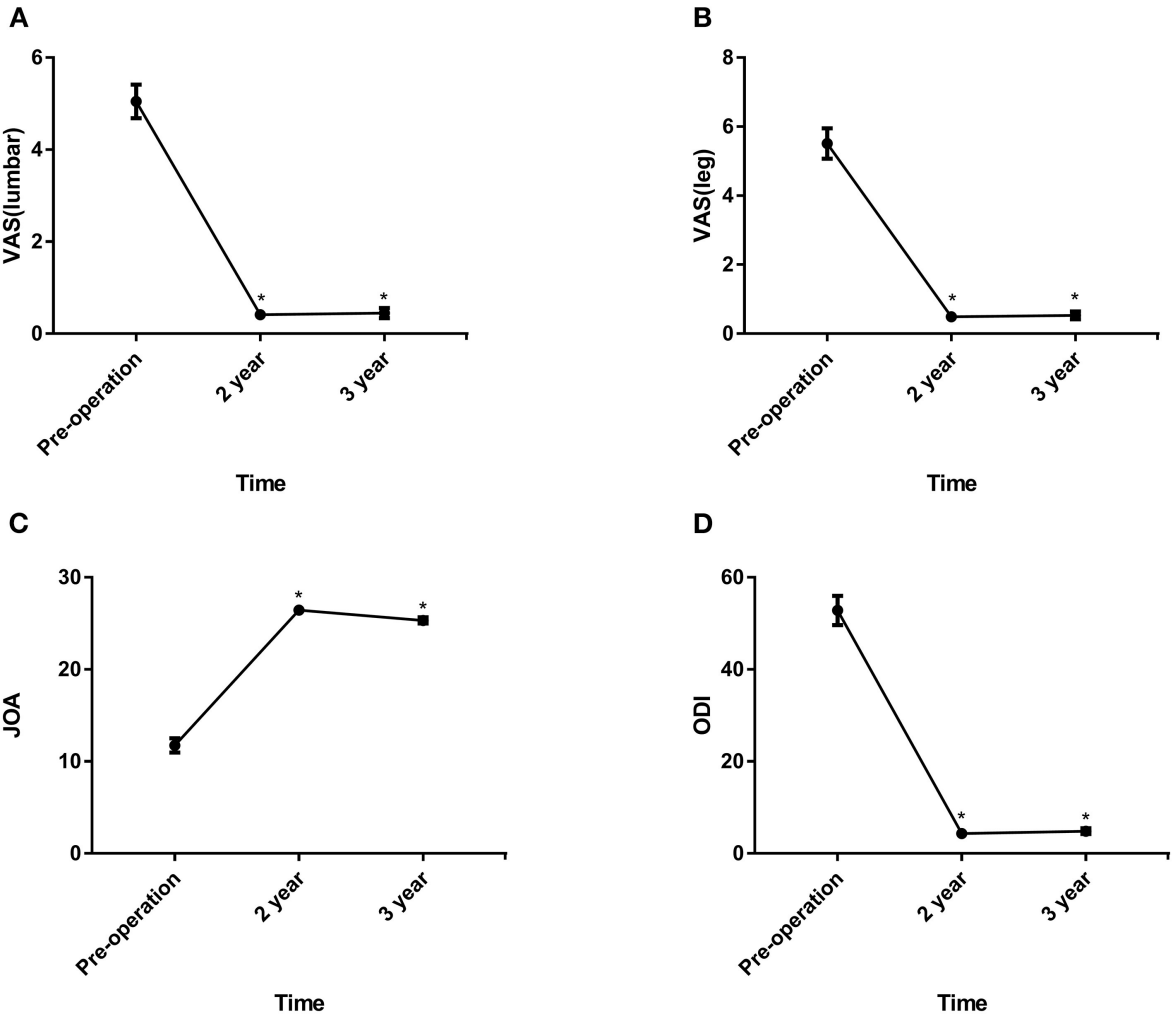
**(A–D)** The VAS (lumbar), VAS (leg), JOA and ODI scores before surgery and at 24 and 36 months postoperatively. VAS, visual analogue scale; JOA, japanese orthopaedic association; ODI, oswestry disability index. *P < 0.01, within-group comparisons between at baseline, 24 and 36 months.

**Figure 3:**
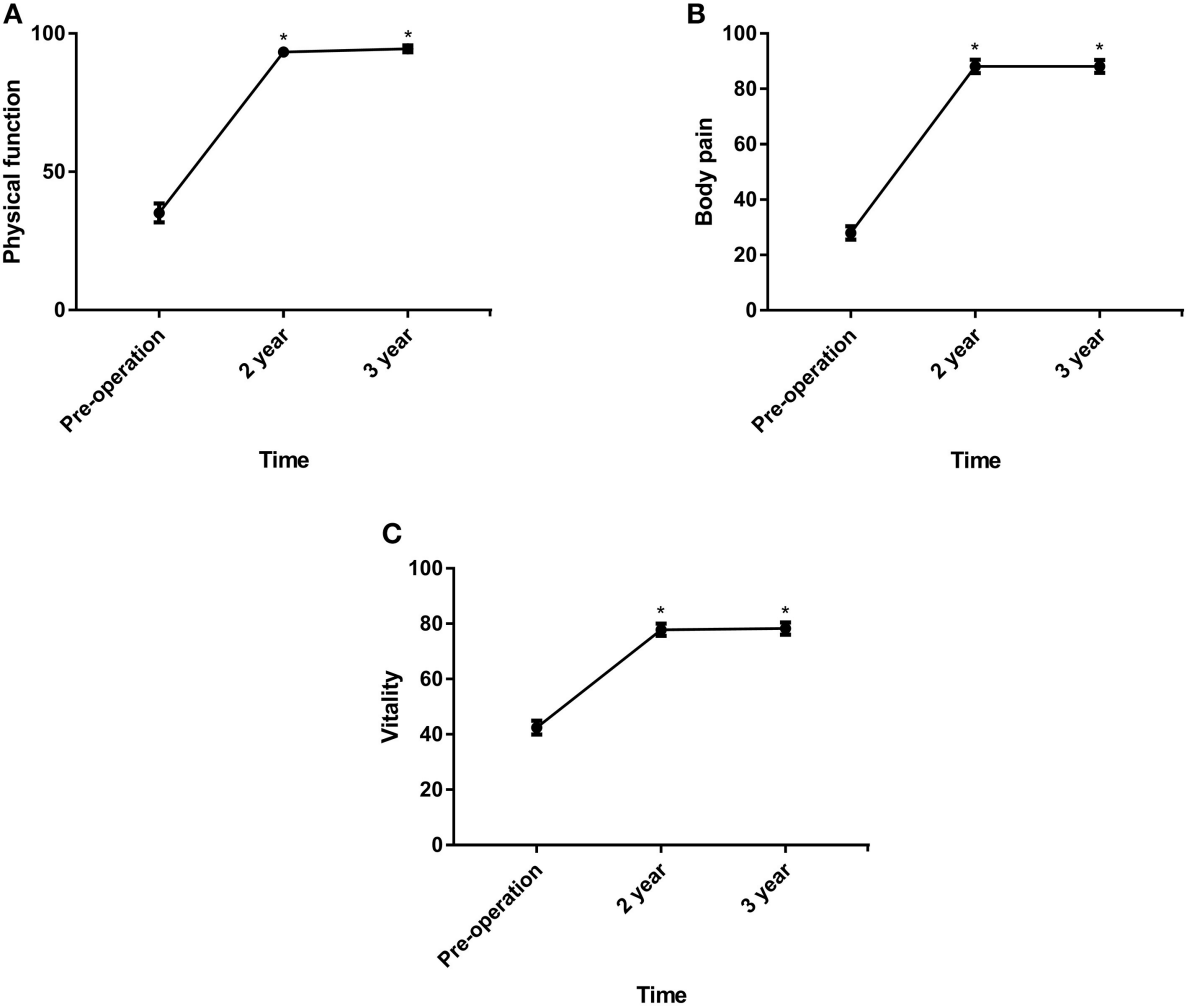
**(A–C)** The SF-36 physical component summary scores (physical function, body pain and vitality) before surgery and at 24 and 36 months postoperatively. *P < 0.01, within-group comparisons between at baseline, 24 and 36 months.

As shown in Figures 2, 3, the VAS and ODI scores significantly decreased from pre- to postoperatively, and the JOA and SF-36 physical component summary scores increased significantly (*P* < 0.05). Comparing the 2-year data, the VAS (lumbar), VAS (leg) and ODI scores increased slightly with significant differences (*P* < 0.05).

Only 1 patient experienced intra-complications (2.4%; dural tear). After 1 week of conservative treatment, the patient exhibited satisfactory recovery. No patients experienced postoperative complications, and 1 patient required reoperation (2.4%) (Table 2).

**Table 2:** Complication and reoperation of included patients.

**Outcome**	**PED**
All complications	1 (2.4%)
Intra-complication	1 (2.4%)
Post-complication	0 (0.0%)
Reoperation	1 (2.4%)

## Discussion

LSS is a common disease that is increasing in frequency in the elderly population worldwide, but it is also common in adults (older than 35–40 years) who commonly perform manual labor and excessively load their spine with heavy loads (22). Conventional laminectomy decompression is a surgical method commonly used for the treatment of LSS (2, 23, 24). The posterior column structure is severely damaged during laminectomy and related facet joint resection, and postoperative complications such as lumbar instability can occur (25, 26). Lumbar interbody fusion is a common treatment for LSS and can prevent lumbar spine instability (27). The resection of joint and soft-tissue structures is also required for the decompression of LSS. With the development of endoscopic technology, it is possible to achieve decompression without destroying these structures (8, 28). However, there is little evidence to support this rapidly developing surgical technique. Therefore, we conducted this study to evaluate the outcomes of PED surgery for LSS. This retrospective trial included 41 patients with LSS, including patients with or without degenerative spondylolisthesis.

The presence of degenerative spondylolisthesis is generally considered a sign of instability, although there is no consensus on the definition of the term (29). Some studies have shown that patients with degenerative spondylolisthesis may be at risk of iatrogenic spondylolisthesis or an increased degree of spondylolisthesis after decompression surgery (30). However, the clinical consequences of spondylolisthesis have been controversial for decades (31). In addition, few studies support the widespread use of fusion surgery in patients with lumbar spinal stenosis, regardless of whether there is spondylolisthesis (32). Despite the lack of a consensus on the definition of instability, surgeons often use decompression and fusion surgery as a means to prevent postoperative instability (29). Two studies have provided the main basis for this fusion surgery (33, 34), but its validity has been questioned (32). The results of a previous study revealed that the clinical efficacy of PED was reliable during a follow-up. A recent study showed that there were no substantial benefits of additionally performing fusion surgery for LSS, even in the presence of spondylolisthesis (29).

Although some studies have reported good clinical outcomes and a low complication rate for endoscopic lumbar decompression, its effect on LSS has not yet been proven. Our study showed that PED also has a satisfactory effect on LSS. In addition, advantages have been reported for PED over traditional surgery, for example, better clinical outcomes, a lower complication rate, a shorter hospital stay, and faster rehabilitation (35, 36). The slow deterioration of surgical results over time has been described, and a similar situation was found in our study at the third-year follow-up (37, 38). Overall, the patients achieved satisfactory results over an average of 3 years of follow-up. Since minimally invasive surgery eliminates the need for the removal of spinal canal structures or reduces the extent of resection, this method seems to reduce the consequences of surgery (39, 40). Postoperative ODI and VAS were significantly improved compared with preoperative values which was similar with previous study (19, 20). Our study showed that 1 patient had dural tear (complication rate of 2.4%). Dural injuries have been reported in the literature to occur at a rate ranging from 0% to ~5% (41–44). In addition, 1 patient required reoperation (2.4%) for incomplete decompression. The reoperation rate is much lower than previous study (16.7%) (20).

To date, it is still difficult to determine well-defined parameters based on evidence-based medicine standards that require fusion in addition to decompression. Some experts have pointed out that patients with predominantly leg symptoms and no signs of segmental instability or deformities should use stability-preserving decompression techniques to avoid fusion (38). Our study revealed that all patients, with or without mild degenerative spondylolisthesis, exhibited satisfactory results. The results were consistent with those of previous studies (23, 29).

This study had some limitations. In this study, the follow-up time was short, and the long-term efficacy of the PED treatment could not be evaluated. Endoscopic techniques have not been used for the treatment of LSS for very long, so the technique is not well-established. Endoscopic decompression still needs to be improved further, and the operation time can be shortened.

In conclusion, the purpose of surgical treatment for LSS is to sufficiently decompress and minimize the trauma and complications caused by surgery. In general, this study did not reveal any obvious shortcomings of PED. Therefore, we recommend PED for LSS. However, prospective randomized controlled trials are needed to verify these results. The cases that require fusion as well as decompression need to be urgently identified.

## Data Availability Statement

The raw data supporting the conclusions of this article will be made available by the authors, without undue reservation.

## Ethics Statement

The studies involving human participants were reviewed and approved by the Clinical Research Ethics Committee of the Tangdu Hospital. The patients/participants provided their written informed consent to participate in this study.

## Author Contributions

F-LW, J-XQ, H-RG, TL, K-LZ, and C-PZ contributed to the revised the work critically. All authors have approved the final version to be published and have agreed to be accountable for all aspects of the work. All authors contributed substantially to the conception and design of the work, acquisition and interpretation of data, and the drafted work.

## Conflict of Interest

The authors declare that the research was conducted in the absence of any commercial or financial relationships that could be construed as a potential conflict of interest.
